# Supplementary figures and images for: Using the Cocrystal Approach as a Promising Drug Delivery System to Enhance the Dissolution and Bioavailability of Formononetin Using an Imidazole Coformer
Source: Pharmaceuticals (Basel). 2024 Oct 28;17(11):1444. doi: 10.3390/ph17111444 (PMC11597446; doi:10.3390/ph17111444)

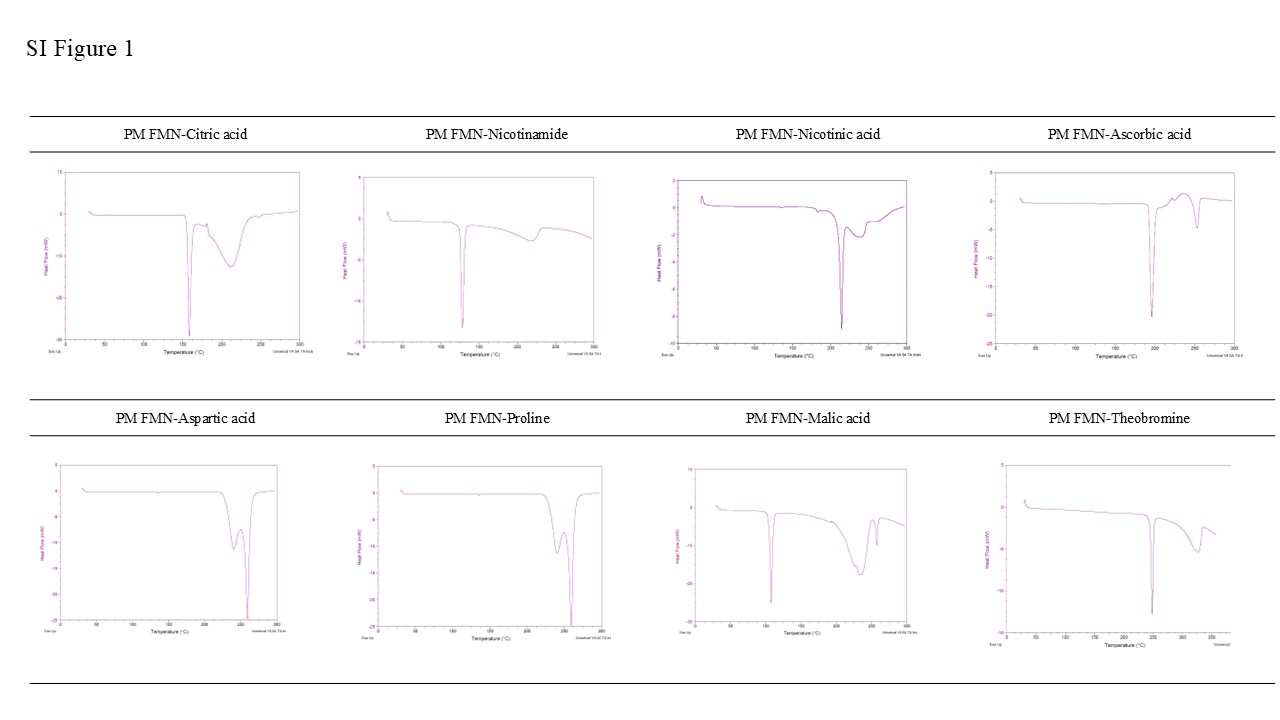

Supplement: Supplementary file 1 [file pharmaceuticals-17-01444-s001.zip › pharmaceuticals-3208697-supplementary.jpg]
